# Spatial Data Starter Kit for OnSSET Energy Planning in Kitui County, Kenya

**DOI:** 10.1016/j.dib.2022.108691

**Published:** 2022-10-26

**Authors:** Daniel Mutia Mwendwa, Jeffrey Tchouambe, Emily Hu, Micaela Flores Lanza, Andrea Babic Brener, Gyubin Hwang, Layla Khanfar, Alycia Leonard, Stephanie Hirmer, Malcolm McCulloch

**Affiliations:** Department of Engineering Science, University of Oxford, Parks Road, Oxford, OX1 3PJ, UK

**Keywords:** Least cost electrification planning, County-level energy planning, GIS, Decentralized energy planning, Energy access, Rural electrification

## Abstract

The 2019 Energy Act requires each of Kenya’s 47 counties to independently develop energy plans. As county energy planning accelerates, it is important to understand the availability and readiness of data required to facilitate it. This article identifies, evaluates, and pre-processes openly available data to facilitate county-level energy planning using the Open Source Spatial Electrification Tool (OnSSET) in Kitui County, Kenya. In this way, it provides a ready-to-use starter kit of data inputs for county-level OnSSET analysis, and guidance to replicate this work in other counties. We classify the readiness level of each data type for county energy planning on a traffic light scale (i.e. green, amber, red) based on availability, accessibility, recency, accuracy, spatial resolution, and format (i.e. whether processing is required before use). Of the 25 core data inputs for OnSSET at the county-level, we find that 14 have a *green*, six have an *amber*, and five have a *red* readiness-level. Data processing requirements are documented, and the processed data for Kitui county are made available as a ready-to-use set of input parameters for OnSSET. While this data was collected for Kitui, the data sources and processing steps are largely applicable in other counties.

**Specifications table**

**Table:** 

Subject	Energy
Specific subject area	Spatial electrification planning and modelling.
Type of data	GIS Files and CSV Tables
How the data were acquired	Literature and database survey; spatial analysis and post-processing.
Data format	Raw and Analyzed
Description of data collection	Publicly available datasets were assessed. Data were collected through an extensive web-search of open access datasets, relevant literature, and governmental reports. No closed-source, commercial, or independently collected data were evaluated. These were evaluated for readiness, analyzed where applicable, and pre-processed to serve as inputs to the OnSSET electrification planning model.
Data source location	• County: Kitui • Country: Kenya • Raw data sources are listed in Table 1.
	• Repository name: Zenodo • Data identification number: 10.5281/zenodo.7075699 • Direct URL to data: www.doi.org/10.5281/zenodo.7075699

**Value of the Data**

• These data can be used to calibrate OnSSET for county-level least-cost electrification planning in Kitui county, Kenya.
• They can be used directly in analysis by academics or policymakers, or serve as a template and guidance on collecting sub-national data as inputs to OnSSET.
• The evaluation of county-level data readiness for OnSSET provides useful insights into data gaps and challenges in decentralized energy planning.
• This is particularly important as energy planning and sustainable development initiatives increasingly move towards emphasis on local, area-based planning, which requires highly resolved ad regionally specific data inputs.
• These data can be used as a starter kit to accelerate teaching of county-level energy planning amongst practitioners and policy-makers.

## 1 Objective

This dataset provides a ready-to-use starter kit for least-cost electrification planning in Kitui County, Kenya. Data identification, evaluation, and pre-processing are documented to serve as guidance for county-level energy planning efforts.

While Kenya had an overall electricity access rate of 71.4% in 2020, this is distributed unequally amongst urban and rural residents (i.e. 94% and 62% respectively) [1], [2] and the country’s 47 counties [3]. To help to address these inequalities, the constitution of 2010 decentralized the responsibility of energy planning to counties [4]. This was compounded by the 2019 Energy Act, which required each county to develop its own energy plan. While most counties are still in the early stages of drafting these plans, efforts are accelerating.

Least-cost electrification planning to inform these energy plans is frequently undertaken using the Open Source Spatial Electrification Tool (OnSSET). OnSSET uses spatial socioeconomic, demographic, climatic, and infrastructural data to evaluate optimal electrification pathways[5]. These data are not always available with high recency, quality, or resolution in under-resourced Kenyan counties. This merits investigation and evaluation, as the resulting plans are necessarily only as robust as the input data used. This brief therefore provides guidance on data availability and quality and an example dataset for this purpose.

## 2 Data Description

The data associated with this work are contained in spatial data files used to create the input for OnSSET in Kitui, and a prepared CSV data input for OnSSET in Kitui (*kitui_OnSSET_data*). The data sources and evaluation process are summarized in Table 1. The following spatial data files are included in the dataset. Further details on each are provided in the following section.

• *kitui_admin*: A vector (.geojson) file containing the administrative boundaries of Kitui county.
• *kitui_clusters*: A vector (.geojson) file locating population clusters generated in data processing for OnSSET.
• *kitui_demand*: A raster (.tif) file containing merged health, agriculture, commercial, and residential demands for Kitui county in kWh.
• *kitui_elevation*: A raster (.tif) containing elevation information.
• *kitui_GHI*: A raster (.tif) file containing global horizontal irradiance data for Kitui county.
• *kitui_hydro*: A vector (.geojson) file containing the locations of hydropower stations in Kitui county. Note that there are none, and that this is expected.
• *kitui_night_lights*: A raster (.tif) file capturing the light emitted from Kitui county at night.
• *kitui_power_stations*: A vector (.geojson) file showing the locations of power stations in Kitui county.
• *kitui_roads*: A vector (.geojson) file showing the main roadways in Kitui county.
• *kitui_transformers*: A vector (.geojson) file showing transformer locations in Kitui county.
• *kitui_transmission_lines*: A vector (.geojson) file locating transmission lines in Kitui county.
• *kitui_travel_hours*: A raster (.tif) file showing travel time to the nearest market center in Kitui county.
• *kitui_wind_100*: A raster (.tif) file of wind speeds at 100 m in Kitui county.

**Table 1:** Summary of the raw datasets identified and pre-processed for OnSSET in Kitui and their readiness level.

Dataset	File Type	Source(s) used	Spatial Resolution	Year	Quality
Population Density/Distribution	Raster	HRSL via HDX[9]	1”	2020	Amber
Administrative Boundaries	Polygon	UNHCR via ArcGIS Hub[7]	N/A	2018	Green
Wind Velocity	Raster	Global Wind Atlas[10]	250m	2018	Green
Solar GHI	Raster	Global Solar Atlas[11]	250m	2017	Green
Travel Hours	Raster	IFPRI via Harvard Dataverse[12]	5’	2010	Green
Elevation	Raster	NASA via WRI[13]	90m	2007	Green
Night-time Lights	Raster	Earth Observation Group[8]	0.0042${}^{\circ }$	2020	Green
Land Cover	Raster	ESA[14]	300m	2015	Green
Substations	Point shapefile	KPLC via EnergyData.Info[15]	N/A	2020	Green
Transformers	Point shapefile	KPLC via EnergyData.Info[16]	N/A	2017	Green
Current HV Network	Line shapefile	KPLC via EnergyData.Info[17]	N/A	2017	Green
Planned HV Network	Line shapefile	No relevant open data found.	N/A	N/A	Red
Current MV Network	Line shapefile	KPLC via EnergyData.Info[17]	N/A	2017	Green
Planned MV Network	Line shapefile	No relevant open data found.	N/A	N/A	Red
Roads	Line shapefile	OpenStreetMap via WFPGeoNode[18]	N/A	2018	Green
Hydropower Stations	Point shapefile	OpenStreetMap via Overpass Turbo[19]	N/A	2022	Amber
Total Population	Float	KNBS Census[20]	N/A	2019	Green
Electrified Population	Float	Earth Observation Group[8] and CIDPs[3]	N/A	2020	Green
Urban Population	Float	FAO via WRI[21] and CIDPs[3]	N/A	2007	Amber
Per Capita Demand	Float	Our World In Data[22], [23], [24]	N/A	2020	Amber
Health Demand*	Raster	ESRI via ArcGIS Hub [25]	300m	2022	Amber
Education Demand*	Raster	Ministry of Education via EnergyData.Info[26]	300m	2020	Amber
Agriculture Demand*	Raster	No relevant open data found.	N/A	2020	Red
Commercial Demand*	Raster	ArcGIS Hub[27], ICPAC[28], UNOCHA via Stanford[29].	300m	2019	Red
Residential Demand Tier*	Raster	Literature-based estimate: [30], [31], [32], [33], [34], [35].	300m	N/A	Red
Gross County Product	Float	KNBS[36]	N/A	2021	Green

*These datasets were generated using the listed sources and various estimation methods detailed in this report.

## 3 Experimental Design, Materials and Methods

This work identifies and evaluated data inputs for least-cost electrification planning in Kitui County, Kenya to form a ready-to-use starter kit of input data for county-level OnSSET analysis. The data inputs needed are shown in the first column of Table 1 [5].

As Kenyan counties are resource constrained, and are expected to produce county energy plans every three years as per the 2010 Constitution and 2019 Energy Act, we consider only publicly available open access datasets. These were sourced through an extensive web-search, thereby avoiding time-consuming and expensive primary data collection. The collected data are evaluated for readiness based on a three point traffic light system, where *green* represents high readiness, *amber* represents fair readiness, and *red* represents poor readiness. Readiness here considers: data availability of data, ease of access, recency, accuracy in relation to other sources, spatial resolution, and format (i.e. whether processing is required before use).

Table 1 shows a summary of the data search and evaluation. Where the data sourcing was not trivial or required analysis or pre-processing, details are provided below.

### 3.1 Administrative Boundaries

Administrative boundaries can be retrieved from a number of sources, such as GADM [6] or the ArcGIS Hub [7]. Different sources agree well on county-level administrative boundaries in Kenya. The ArcGIS Hub hosts data from the Field Information and Coordination Support Section of the Division of Programme Support and Management at UNHCR; given the high credibility of the source, these data are used and the readiness-level is considered as *green*.

### 3.2 Total, Electrified, and Urban Population

OnSSET requires input data on total population, the proportion with existing energy access, and relative urbanity. A number of data sources, including the 2019 census, the World Bank, and UNDESA give similar values of population at national-level. However, at county-level, there are fewer sources for population figures. One useful source is the Council of Governors (CoG) county integrated development plans (CIDPs) [3] which disaggregate many census results at county level. They are available at county-level across all counties, and provide several types of population data types needed in OnSSET (e.g., electrified population, urban population, etc) in one report, making them a convenient data source which can be used in Kitui and other counties. Alas, the CIDPs do not provide these data in GIS-ready formats; so, while the statistics for population there can be used to cross-check alternative sources, they cannot be used on their own. Given this, rasters of electrified population can be sourced from night-time lights data, including the Earth Observation Group night lights dataset [8], which are ranked *green* given their recency, availability, and high resolution.

This is extracted as part of the data processing and clustering process documented later in this paper. Urban population data are extracted from the FAO Africover dataset, via WRI [21]. Given the age of this dataset (2007), it is classified as *amber*.

### 3.3 Population Density and Distribution

Population density and distribution data can be retrieved as a raster from a number of sources, including WorldPop [37], the Gridded Population of the World (GPW) datasets [38], and the High Resolution Settlement Layer (HRSL) [39], available in its most up-to-date form on the Humanitarian Data Exchange (HDX) [40]. These datasets are all highly reputed. However, the highest resolutions available for WorldPop and GPW is 30 arc-seconds (i.e. approximately 1 km at the equator), whereas the highest resolution available in HRSL is 30 m. Higher resolution data can enable better population clustering in the OnSSET model; as such, the HRSL data from the HDX is selected. These data are also preferred as they are easy to access online in OnSSET-ready formats. The HRSL dataset is rated *amber* as it is not empirical. It is based on ground-truth sources, but machine learning and modelling are then used to produce the final population dataset [41]. As such, it represents a good approximation of population distribution but is not exact. Indeed, all three data sources used to incorporate some degree of modelling to fill gaps.

### 3.4 Renewable Resource Potentials

To evaluate generation options, OnSSET requires data on the spatial distribution of wind and solar potential.

*Wind* speed information can be obtained from Global Wind Atlas [10] or Renewables.Ninja [42]. The Global Wind Atlas dataset covers onshore areas globally, and up to 200 km of offshore areas extending from the shoreline. The horizontal grid spacing is 250 m, wind speed is available at 10, 50, 100, 150, and 200 meters above ground level. According to the dataset documentation, is constantly validated with several relevant sources [43]. Renewables.Ninja offers information about wind speeds that are converted into power output using the Virtual Wind Farm (VWF) model [42]; however, this data cannot be easily downloaded in a raster form. Here, the Global Wind Atlas is used because it is highly accurate (i.e. constantly updated and verified and discussed above) and easy to use (i.e. available online for download as a raster, unlike Renewables.Ninja). For these reasons, it is given a *green* readiness-level ranking.

*Solar* global horizontal irradiation (GHI) data is similarly available from Global Solar Atlas [11] and Renewables.Ninja [42]. The grid resolution of Global Solar Atlas solar resource data is between 3 to 7 km, depending on the latitude; however, it can be enhanced by down-scaling the grid to a nominal resolution of approximately 1 km. Also, the spatial resolution of other parameters has been harmonized to 1 km. This source covers areas between latitudes 60${}^{\circ }$N to 45${}^{\circ }$S. The areas north and south of these coordinates are not covered due to the incline of the satellite imagery does not accurately assess cloud cover. These data have been validated at more than 228 sites worldwide using more than 20 global networks. Renewables Ninja [42] also offers information about solar irradiation, with information from 2019 that is converted into power output using the GSEE model (Global Solar Energy Estimator). Here, the Global Solar Atlas is used because it is highly accurate (i.e. constantly updated and verified and discussed above) and easy to use (i.e. available online for download as a raster, unlike Renewables.Ninja). For these reasons, it is given a *green* readiness-level ranking.

### 3.5 Grid Infrastructure

Existing grid infrastructure is taken as an input in OnSSET to determine where grid extension is more feasible as compared to stand-alone systems. Input data requirements on grid infrastructure include substations, transformers, high-voltage (HV) and medium voltage (MV) lines, and existing hydropower plants.

*Substations* are located and validated using multiple publicly-available data source with the following method. Firstly, Kenya’s substations are obtained from Energy Data [15], where a dataset in GeoJSON format provided by Kenya Power and Lighting Company (KPLC) is available. These locations are cross-checked with the International Renewable Energy Agency (IRENA) interactive map of Kenya [44] which provides the locations of the substations with their respective maximum rating. Finally, Kenya’s power line information is obtained from Overpass Turbo [19] by developing an API query for ǣpower=lineǥ. The resulting GeoJSON dataset is downloaded and used in QGIS to validate the intersection between the previously downloaded power lines and substations. This cross-validation between data sources provides a high-confidence (i.e. *green* readiness-level) dataset.

*Transformers* are located using a similar process. Transformer locations are initially obtained from Energy Data [16] as a GeoJSON dataset, and then these locations are compared with the points where transmission lines change voltage according to IRENAs interactive map of Kenya [44]. Finally, transformer locations are validated by running API queries on Overpass Turbo [19] of power lines with different voltages, downloading the respective GeoJSON files and comparing the locations where these changes occur with the transformer locations. Using this process, the datasets were found to agree, indicating a high-quality dataset with readiness level *green*. Note that KPLC has also published a report with the location of the transformers by county; however, this document only provides the description of the locations rather than the coordinates, and therefore the above method of collecting the data is suggested [45]. This KPLC document can still be used as a cross-validation source if desired.

*Hydropower plants* are identified as follows. First, using the Wizard Tool in Overpass Turbo [19] and inserting the query plant:source=hydro, the specific points where hydropower plants are located are obtained. To validate this dataset, the location of the hydropower plants provided by the query is compared with satellite imagery from Google Earth. This dataset from Overpass Turbo effectively matches visible hydropower plants on Google Earth. Interestingly, the results of a different query generator:source=hydro provided by Overpass Turbo did not match the satellite imagery from Google Earth. This example elicits the need to cross-check data sources when looking for data. Given this discrepancy, the data are ranked at an *amber* readiness-level.

*Current HV and medium-voltage MV networks* are available through EnergyData.Info and provided by KPLC [17]. These data were collected in preparation for the Kenya Off-grid Solar Access Project and the Kenya National Electrification Strategy, launched in December 2018. These data have a *green* readiness level based on their recency (i.e. they are from 2017), ease of access (i.e. available online in OnSSET-ready formats) and source credibility (i.e. they are uploaded to EnergyData.Info by KPLC).

*Planned HV and MV networks* were not found in any publicly available data source, and so these data points have a *red* readiness level.

### 3.6 Demand

To model systems of appropriate sizes, input data on expected demands are required. This can be broken down into demand per capita, and sectoral demand in health, education, agriculture, and commercial applications. A residential demand tier can also be specified for new connections, and gross county product (GCP) or gross domestic product (GDP) per capita can be used to scale demand estimations.

#### 3.6.1 Per Capita Demand

Primary energy consumption data in Kenya varies greatly based on the source consulted. For instance, primary energy consumption in Kenya was estimated as 1,906 kWh in 2016 by Our World in Data [22], and 146.23 kWh in 2018 by WorldData. It is often unclear whether different sources report primary, final, or electricity-only energy consumption, adding to this confusion. Electricity demand per capita in Kenya is given by the International Energy Agency until 2014, but this is at this point beginning to fall out of date.

Energy demand per capita data from Our World in Data are used here. These are based on the BP Statistical Review of World Energy [23] and the Shift Energy Data Portal [24]. While BP provides the most up-to-date values of primary energy, it does not provide data for all countries, so data from the Shift Energy Data Portal are used to fill gaps. The Shift Project, in turn, draws upon information from World Energy Production [46] and US EIA Historical Statistics [47]. To convert from total primary energy to per capita energy, population figures from the UN World Population Prospects [48] were used.

This dataset has several limitations. While it is a best effort to create full coverage using different data sources, these sources may adopt different definitions for energy use and have different qualities. Furthermore, several figures used in these calculations to create the final dataset are outdated, and distinctions between electricity and energy is absent in some sources. Finally, the trends in growth do not agree between some sources from Our World in Data and WorldData. Given these issues, the data used are given an *amber* readiness-level.

#### 3.6.2 Health Demand

No publicly available empirical data sources about the spatial variation of health energy demands were found in this search. These demands were therefore estimated using the following method. Healthcare demand can be assumed to be proportional to demand for health care services, which can in turn be assumed to be proportional to the spatial distribution of healthcare facilities. As such, a data source mapping of healthcare facilities is first sourced to facilitate demand estimation. Available data sources for this are listed in Table 2. As evident in Table 2, different datasets have different quantities of operational facilities, which indicates that there may be accuracy issues.

**Table 2:** Datasets found on Kenyan medical facilities.

Source	Number of Operational Facilities
Kenya Master Health Facility List [49]	10,000
Kenya Medical Practitioners and Dentists Council [50]	6,039
ArcGIS [25]	4,867
World Bank [51]	10,013

The work of Franco [52] provides two different methodological starting points for developing a raster for health demand based on health facility locations:

a) Energy demand can be estimated from the bottom up based on the equipment owned by different healthcare facilities. and the power ratings for different types of equipment (as provided in [52]).
b) Alternatively, demand can be estimated based on different health facility types. If healthcare facility data are classified into standard types, a set of standard demand profiles can be applied across all healthcare facilities of each type. Franco suggests demand estimates for four health facility types (i.e. hospitals, health centers, health clinics, and health posts) as captured in Table 3.

**Table 3:** Energy consumption of varying types of health facilities, from [52].

Facility type	Size	Consumption	Peak Demand
Hospitals	Over 120 beds	1535 kWh	9 kW
Health centers	60120 beds	10 to 20 kWh	5 kW
Health clinics	0 to ±60 beds	4 to 10 kWh	2.4 kW
Health posts	Functions As Storage	N/A	N/A

Based on the health facility data data available, one of these two approaches must be selected. From the sources in Table 2, the Kenya Master Health Facility List, Kenya Medical Practitioners and Dentists Council, and World Bank data sources did not list the types of health facilities or the equipment contained in each. Additionally, some of these were difficult to extract from web interface. While the ArcGIS dataset had less datapoints than the others, each data point was categorized by type, which facilitated demand estimation using approach b, and the data was available and easy to extract in an OnSSET-ready spatial format [25]. As such, these data were selected for use here. However, given the discrepancies with other data sources, this dataset was given an *amber* readiness-level ranking. Using the selected vector file of health facilities across the county of Kitui and their classification, demand was assigned based on the demand estimates in Table 3. Given the amber ranking of the underlying health facility data, the resulting demand estimation is also ranked at an *amber* readiness level.

#### 3.6.3 Education Demand

Education demand is estimated in a similar way to health demand. First, education facilities are identified through a data search; then, demand is assigned to each based on best available esitmates. A search was made for datasets counting and locating schools in Kenya. Different data sources showed different quantities of schools, as evident in Table 4. A vector dataset of primary and secondary school locations published in 2017 by the World Bank has approximately 37,930 entries, while a dataset created as part of the Kenya School Mapping Project encompassing kindergarten, primary, secondary, and universities estimates about 73,000 educational facilities [53].

**Table 4:** Datasets found on Kenyan educational facilities.

Source	Number of Operational Facilities
Kenya School Mapping Project [53]	73,000
World Bank [26]	37,930

Unfortunately, data from the Kenya School Mapping project is currently inaccessible online. However, it may be possible to obtain this data by contacting the paper’s authors – this can be explored if desired in future efforts. In this work, given the desire to use publicly accessible data, the World Bank dataset was used.

While there is little empirical data to be found on energy use by educational facilities in Kenya, some estimates on consumption can be found in the literature (e.g. Moner-Girona et al [54]). This, alongside the number and locations of facilities, was used to estimate demand. The resulting data are assigned a readiness-level of *amber* given the discrepancies in the sources for the quantities of schools and the lack of detail in the demand estimation applied across schools.

#### 3.6.4 Agriculture Demand

Agriculture is a large part of the Kenyan economy and is important to consider when modelling energy demand. However, agricultural electricity demand data in Kenya is not readily available at the national level, let alone at the county levels. Statistica provides the value of fuel and power consumed in agriculture in Kenya from 2016 2020 [55]; however, this data is not publicly available without registration for the service. As such, it is ranked *red* for readiness. Information regarding land available for agricultural activity from the CoGs county level documents [3], or total agricultural products produced in the country [56], could be used alongside estimations of electricity needed for different types of crops (e.g. from the University of Warwick [57]) to estimate demand. This is not expected to be highly accurate however, given the layers of estimation involved and a lack of spatially disaggregated data. Moreover, a wide range of technologies are used by Kenyan farmers, from small-scale, residential manual labour to larger, mechanised irrigation. Agricultural energy use is split amongst multiple energy vectors (e.g. manual labour, petrol, electricity), making it difficult to isolate electrical demands specifically. Though rural levels of electrification are low, studies have shown that rural electrification is significantly associated with higher agricultural output [58]. How much electricity used is directly related to the type of farming methods employed; no data is available on the prevalence of different farming methods in different Kenyan counties, which further complicates demand estimation.

Given these difficulties, no viable estimate for agricultural demand was found. It is assumed that electrical energy demand from agriculture will be included in commercial or residential demand; non-electrical energy demands from agriculture are not modelled here. In the future, to get better, more accurate estimates of agricultural electricity demand, it is recommended to add a section to census data or surveys to better understand this, and to document the technologies used for agriculture by households.

#### 3.6.5 Commercial Demand

No data source was found to assess spatially disaggregated commercial demands in Kitui county. As such, an estimate was generated based on available data. Rather than focus on commercial demands of all kinds, we generate a commercial demand estimate with a focus on enabling markets: cell towers, warehouses, and bank data. The potential demand arising from the electrification of water pumps is also considered under the assumption that certain economic activities may occur around water sources, including irrigation. Data used to generate this demand estimate included ArcGIS Hub data on cell phone towers and banks [27], ICPAC Geoportal data on waterpoints [28], and Stanford Digital Repository data on warehouses [29]. Interestingly, at the county level, there are noticeable differences between some enabling services found in the data and those reported by counties in their integrated development plans, suggesting that the data may be incomplete or inexhaustive. To verify this, county governments can be queried for data, particularly regarding waterpoints and warehouse locations.

Data was sparse on standard energy demands for these enabling services in the Kenyan context. As such, standard demand estimates from other contexts are used. Bank data is obtained from Yurtsever et al [59], which shows that energy use in bank branches varies insignificantly from one to another, using the example of a Turkish bank. Despite the location, in the absence of Kenya-specific data, the average monthly consumption identified was ascribed to bank data found in Kenya. Cell tower consumption is obtained from a paper by Owino [60], assessing the use of renewables to power a base station in Sekanini, Kenya. Little information is given to discuss how this varies with the size of the base station. For this work, the given demand is used for all identified cell towers. Warehouse data is also obtained from Lewczuk et al (2021) [61]. This is based on industrial warehouses in Poland; thus, the lower bound is applied to warehouses identified in Kenya in the Stanford digital repository. Waterpoint demand assumes a $\frac{1}{4}$ horsepower pump is adequate as per information from an environmental equipment supplier [62].

The data sources and methods are not exhaustive and understate actual needs. Additionally, demand estimates from other countries are used here to construct a demand estimate for Kenya. As such, the resulting commercial demands dataset is assigned a *red* readiness level. An alternative and potentially more accurate way to evaluate commercial demand would be to request data from local governments on the locations and types of registered formal businesses within their jurisdictions. This assumes that businesses complete registration processes, which may not be the case, and that the addresses of said business are also collected in registration, from which coordinates can be determined and subsequently a shapefile developed. An assessment of demands for each business, however, will still be challenging unless empirical data on commercial demands in Kenya is collected.

#### 3.6.6 Residential Demand Tier

There is little in the way of data to determine a residential demand tier input. This can, however, be estimated at the very least for urban areas using the following approach. The accuracy of this estimation is debatable, but it offers a starting point for creating a raster data input for OnSSET.

To estimate residential demand tier, relationships between between spatial variations in income and energy demand are first investigated. We found two sources assessing the income elasticity of energy demand in Kenya with different long-run elasticities, namely 0.001 [30] and 0.1 [31]. In the absence of a clear consensus, either of these elasticities may be used; further research is required to nail this down. Here, 0.1 is used.

**Fig. 1:**
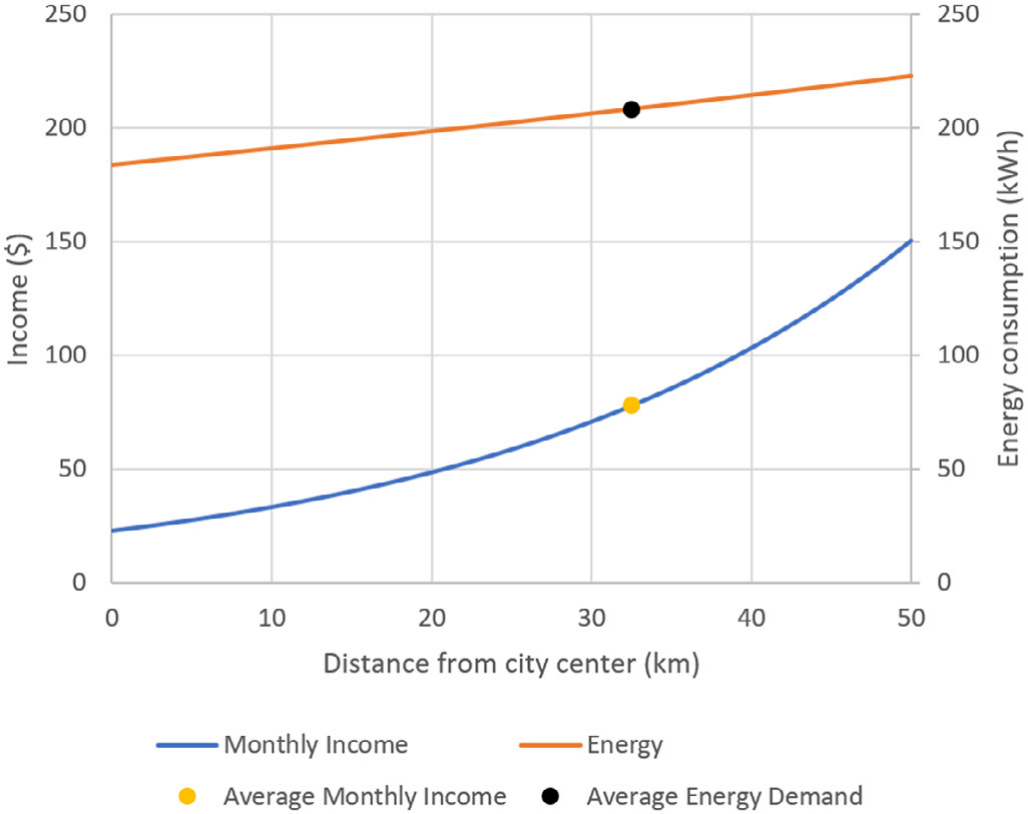
Change in income and energy demand based on distance from city center.

These values can be used to scale residential consumption across urban areas based on the relationship between incomes and geographic factors. In a study based on British cities, Cuberes et al. establish that, in general, income grows the further away from the central business district (CBD) an individual or household is, up to 50 km from the urban center [32]. A 10% increase in income results in a household located on average 2.6% further from the city center. Other factors do play a part in this; however, to generate an OnSSET input, and for simplicity, these were not considered. By taking monthly income at a rate of $1.9 a day (the international poverty level) and assuming that to be the average income at the CBD, one can plot the change in income as distance from the CBD increases, whilst at the same time plotting change in annual energy demand, as demonstrated in Figure 1.

The average national income and household energy demand were found to be $76/month [33] and 208 kWh [34] respectively. It was assumed that this demand corresponds with this income; this allows energy demand across different incomes to be extrapolated based on the variability of demand with distance from CBD. The World Resource Institute provides a data set of major urban centres in Kenya [35]. This vector data was used in conjunction with building data obtained via OpenStreetMap to generate a raster map of urban residential energy demand. Given the UK-based data used to generate this estimate, and the assumptions involved in this process, this data is assigned a *red* readiness level.

#### 3.6.7 Gross County Product

Information on Gross County Product (GCP) can be found at the Kenya National Bureau of Statistics [36] for the 47 counties, divided into 17 economic activities. The latest information is available in the 2021 GCP Report. Given that Kenyas National Bureau of Statistics issued the first GCP Report in 2019 and the second one in 2021, it is expected that updated information will be widely available every two years. This frequently updated data source from a reputed institution is assigned a *green* readiness level. Another approach to get similar data would be to obtain Kenyas GDP data from the Central Bank of Kenya [63] and estimate each countys contribution using the countys share of GDP from the latest GCP Report.

### 3.8 Data Processing

**Fig. 2:**
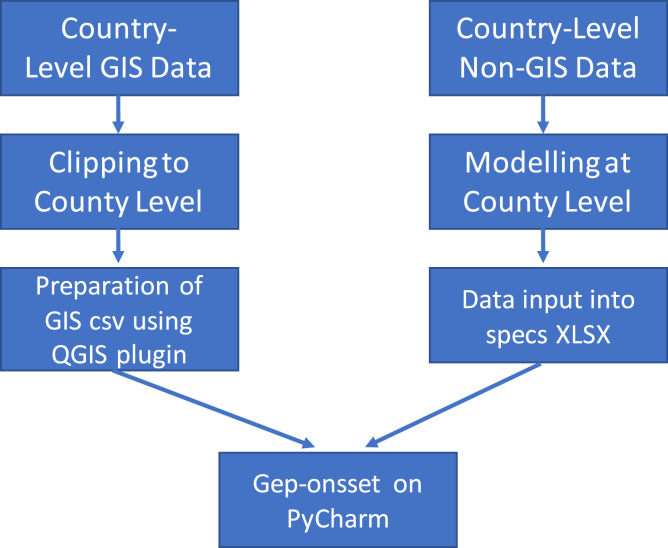
Data Processing Workflow

The collected data are then processed for OnSSET. This involves the preparation of both GIS and non-GIS data to produce a comma-separated values (CSV) file, which can then be fed as the input into OnSSET as shown in Figure 2.

First, any data obtained at a national or regional extent was first cropped to county-level. This involved using the QGIS tools for ǣextracting layer by extentǥ for vector layers and ǣclipping raster by mask layerǥ for raster datasets. County administrative boundaries were used as the mask layers in the cropping.

Population clusters, the basis of the OnSSET analysis, were then generated using the HRSL Clustering plugin developed by KTH which is available on Github [64]. The main challenge in clustering was finding the best projection system for input data. Here, EPSG:21037 (i.e. UTM zone 37S) is used. However, if replicating this process, it is recommended to use the appropriate projection for your area of interest from the WGS84 datum, which is the reference system for global positioning system (GPS). The regions covered by different projections can be found by searching on the EPSG coordinate systems website [65]. The inputs for the clustering plugin were a population raster file, county administrative boundary, and night-time lights. The clustering for Kitui county resulted in 47,660 clusters. Adopting the same data quality scale as above, the clusters are rated as *amber* as the night-time lights data used to generate them were from 2016, and therefore did not capture the electrification rate in our base year (2020) well. However, up to date night-time data is now available [8] and could be used to generate better clusters in future work.

The data were then aggregated in a CSV input file for OnSSET. It was attempted to generate the OnSSET CSV input file using the GEP_OnSSET Plugin developed by KTH-dES which is available on Github [66]. The inputs to the this process included population clusters developed in previous stage, as well as the solar GHI, wind speed, travel hours, elevation, landcover, existing MV & HV transmission lines, roads, substations and transformers data sourced previously. However, the plugin did not produce a final CSV as expected. It instead generated a shapefile with mean values for various parameters, which could then be imported as a CSV. The rest of the parameters were generated by placing them on relevant cluster centroids using the ǣjoin attribute by locationǥ feature on QGIS. The quality of the CSV input file is similarly rated as *amber*. This is because: 1) the clusters used had a poor reflection of the electrification rate, due to the use of 2016 data; 2) the travel hours dataset used was self-generated (i.e. was not the dataset actually listed in the table) and might have inaccuracies; 3) data on demand for various sectors such as agriculture, schools, hospitals and commercial activities was hard to find, as previously documented; and 4) some information might have been lost by using the “join attribute by location” feature to read data into clusters, which picked values at centroids instead of doing averages. Improvements can be made by using up to date night-time lights data, using updated travel hours dataset available e.g. [12], developing more up to date energy demand datasets and improving the compatibility of the OnSSET plugin to ensure batch processing of datasets under the same rules.

**Ethics Statements**

This work did not involve human subjects, animal experiments, or data collected from social media platforms. All raw data are available under open licenses.

## CRediT authorship contribution statement

**Daniel Mutia Mwendwa:** Software, Validation, Formal analysis, Investigation, Writing – original draft, Writing – review & editing, Project administration. **Jeffrey Tchouambe:** Validation, Investigation, Writing – original draft, Writing – review & editing. **Emily Hu:** Validation, Investigation, Writing – original draft, Writing – review & editing. **Micaela Flores Lanza:** Validation, Investigation, Writing – original draft, Writing – review & editing. **Andrea Babic Brener:** Validation, Investigation, Writing – original draft. **Gyubin Hwang:** Software. **Layla Khanfar:** Investigation. **Alycia Leonard:** Writing – original draft, Writing – review & editing, Supervision. **Stephanie Hirmer:** Funding acquisition, Conceptualization, Writing – review & editing. **Malcolm McCulloch:** Funding acquisition, Conceptualization, Methodology, Supervision.

## Declaration of Competing Interest

The authors declare that they have no known competing financial interests or personal relationships that could have appeared to influence the work reported in this paper.

## Data Availability

Spatial Data Starter Kit for OnSSET Energy Planning in Kitui County, Kenya (Zenodo).
